# Heterologous overexpression of *heat shock protein 20* genes of different species of yellow Camellia in *Arabidopsis thaliana* reveals their roles in high calcium resistance

**DOI:** 10.1186/s12870-023-04686-x

**Published:** 2024-01-02

**Authors:** Lisha Zhong, Yuxing Shi, Shaolei Xu, Sisi Xie, Xinhui Huang, Yujie Li, Chaofan Qu, Jianxiu Liu, Jialin Liao, Yang Huang, Yu Liang

**Affiliations:** 1 Key Laboratory of Ecology of Rare and Endangered Species and Environmental Protection, Guangxi Key Laboratory of Landscape Resources Conservation and Sustainable Utilization in Lijiang River Basin, College of Life Science, Guangxi Normal University, Guilin, China; 2 School of Mechanical and Electrical Engineering, Guilin University of Electronic Technology, Guilin, China

**Keywords:** *Camellia Limonia*, *Camellia Nitidissima*, *Arabidopsis thaliana*, High calcium stress, *Heat shock protein 20*, Transcriptome

## Abstract

Yellow Camellia (*Camellia sect. chrysantha*) is a rare ornamental plant and an important germplasm resource globally. *Camellia nitidissima* thrives in normal acidic soils, while *Camellia limonia* can adapt to the calcareous soils found in karst areas. Our previous study on the karst adaptation of yellow camellias revealed that the expression levels of *heat shock protein 20*(*HSP20*) were higher in *Camellia limonia* than in *Camellia nitidissima*. However, the functions of the *HSP20* gene of *Camellia limonia* remain unclear to data. In this study, the *HSP20* genes of *Camellia limonia* (ClHSP20-OE lines) and *Camellia. nitidissima* (CnHSP20-OE lines) were cloned and overexpressed heterologously in *Arabidopsis thaliana*. Additionally, we overexpressed the *HSP20* gene of Arabidopsis (AtHSP20-OE lines) was also overexpressed, and the T-DNA inserted mutants (athspmutant lines) were also used to determine the functions of *HSP20* genes. Under high calcium stress, the chlorophyll, nitrogen, water content and humidity of leaves were increased in ClHSP20-OE lines, while those of other lines were declined. The size of the stomatal apertures, stomatal conductance, and the photosynthetic efficiency of ClHSP20-OE lines were higher than those of the other lines. However, the accumulation of H_2_O_2_ and O_2_^−^ in the leaves of ClHSP20-OE lines was the lowest among all the lines. Energy spectrum scanning revealed that the percentage of calcium on the surfaces of the leaves of ClHSP20-OE lines was relatively low, while that of athspmutant lines was the highest. The *ClHSP20* gene can also affected soil humidity and the contents of soil nitrogen, phosphorus, and potassium. Transcriptome analysis revealed that the expressions of *FBA5* and *AT5G10770* in ClHSP20-OE lines was significantly up-regulated compared to that of CnHSP20-OE lines. Compared to that of athspmutant lines, the expressions of *DREB1A* and *AT3G30460* was significantly upregulated in AtHSP20-OE lines, and the expression of *POL* was down-regulated. Our findings suggest that the *HSP20* gene plays a crucial role in maintained photosynthetic rate and normal metabolism by regulating the expression of key genes under high-calcium stress. This study elucidates the mechanisms underlying the karst adaptation in *Camellia. limonia* and provides novel insights for future research on karst plants.

**Supplementary Information:**

The online version contains supplementary material available at 10.1186/s12870-023-04686-x.

## Introduction

Yellow Camellia (*Camellia sect. Chrysantha*) is an evergreen shrub or small tree belonging to the Theaceae family. Due to the scarcity of germplasm resources and high ornamental value, it is often referred to as the “giant panda in the plant world” and “the queen of the tea Clan” [1]. According to the List of Wild Plants under State Key Protection published in August 2021, all species of *Camellia sect. Chrysantha* are second-class wild plants under state protection [2]. Yellow Camellia possess significant medicinal properties, including anti-tumor, anti-oxidative, anti-inflammatory, and anti-allergic activities, and also aid in the prevention and treatment of three high levels, anti-inflammatory and anti-allergy [3]. More than 20 species of *Camellia sect. Chrysantha* have been reported to date that are mainly distributed in the Guangxi Zhuang Autonomous Region, southern China, and northern Vietnam [4]. Yellow Camellia can be broadly categorized into two types based on their growth environment. One type of camellias is found in the mountainous limestone region of karst areas (stony mountainous type), while the other type grows in normal soils (earthy mountainous type) [5]. The yellow Camellia that grow in karst soils are calcium-resistant and grow well in high-calcium environments. Seven species of yellow Camellia, including *Camellia nitidissima, Camellia indochinensis*, *Camellia tunghinensis* and *Camellia euphlebia*, grow in normal soils, whereas species thirteen species of yellow Camellia, such as *Camellia limonia*, *Camellia pingguoensi* and *Camellia impressinervis*, are the karst type species. *C. limonia* grows in calcareous soil in karst rocky mountainous areas, while *C. nitidissima* can only survive in normal soil in mountainous areas, and it grows slowly or even dies in calcium-rich environment [6]. Notablt, there is no species of any yellow Camellia has been found to grow both in karst limestone mountain and normal earth mountain areas. This indicates that there are definite differences among these species of yellow Camellia species, and suggests that the karst adaptation developed in the karst type species during evolution. However, the mechanism underlying the karst adapatation of yellow Camellia remains unclear, and the functions of special genes that mediate karst adaptation in karst type yellow Camellia require further investigation.

The karst ecosystem accounts for 15% of the world’s land area [7]. One of the most significant geographical features of karst landforms is high-calcium levels, and the average calcium content in the soil is several folds higher than that of non-karst regions [8]. Calcium is an essential nutrient for plant growth and also mediates cellular signal transduction, and is therefore an important signaling molecule in the environmental stress response of plants [9]. Calcium primarily exists in the Ca^2+^ form in soils and functions as a regulatory factor in plant growth and development, and is thus involved in almost all aspects of plant growth and development [10, 11]. However, high concentrations of soil calcium can be toxic to plants and affect their growth; therefore, a relatively suitable concentration of calcium should be maintained in the cytoplasm of plant cells [12]. When the content of soil calcium is very high, plants absorb very high levels of calcium that exceed normal levels and have several harmful effects, including the hardening of plant cell walls, inhibition of cell growth, disorders in phosphoric acid-based energy metabolism, and a reduction in photosynthesis and rate of transpiration, which leads to leaf senescence [13, 14]. Therefore, studies on high-calcium stress can provide better insights into the abiotic stress response of plants and the regulation of plant growth.

At present, the characteristics of karst plants are being investigated in some studies. It has been reported that *Lonicera japonica* Thunb. plants growing in karst areas have certain morphological characteristics that enable them to adapt to karst regions. Additionally, these plants can maintain the stability of the green membrane system and structure and improve the activity of the enzyme protection system by regulating osmotic pressure and changing photosynthetic rate [15]. A previous study observed that the thickness of the leaves, thickness of the upper and lower epidermis, and the degree of compactness of the palisade tissue of *Vitex negundo* growing in karst soils are much higher than those of plants growing in non-karst areas [16]. Studies on the mechanisms of adaptation of *Lonicera Confusa* have revealed that the absorbed calcium is transported to the glands, epidermal hairs, and stoma of leaves in high-Ca^2+^ environments. Previous studies have demonstrated that plants growing in karst regions have their own mechanisms of adaptation to varying soil conditions, irrespective of whether they are adapted to soils with high or low calcium levels [8]. The present study aimed to determine the calcium content and adaptability of plants in karst regions to high-calcium levels, and the findings revealed that the calcium content in the aboveground parts of all functional groups was significantly higher than that of the underground parts of the plants (*p* < 0.05) [9]. These observations suggested that the dominant plants in karst regions employ different mechanisms for adapting to the high-calcium stress resulting from the high soil calcium levels.

Studies on the mechanism of adaptation of plants to calcium stress are of immense significance for a better reconstruction of the ecosystem of rocky desertified areas in karst regions and for selecting suitable plant species for restoring the vegetation. *Camellia limonia*, which is suitable for karst regions, serves as a valuable resource for studying the mechanism of adaptation of plants in high-calcium environments. According to our previous studies, the adaptability of *C. limonia* to high-calcium environments is superior to that of *C. nitidissima*, as revealed by metabolomics, transcriptomics, and metagenomic analysis of the rhizospheric soils of the two yellow Camellia. Comprehensive analysis of metabolomics, and metagenomics analyses revealed that several key genes are involved in regulating the karst adaptation, of which the expression of the *heat shock protein 20* (*HSP20*) gene is has higher in the karst species, *C. limonia* than in the non-karst species, *C. nitidissima.* This suggested that the *HSP20* gene may play a role in the resistance of *C. limonia* to get resistance in high-calcium stress [17].

HSPs are a class of proteins that can undergo rapid synthesis in response to environmental stress and has the function of companion [18]. It has been reported that the *HSP20* gene is involved in the abiotic stress response of plants. Studies aimed at identifying the *HSP20* gene of in *Cucurbita moschata* and *Malus pumila* revealed that the *HSP20* genes are highly induced by heat stress [19, 20]. Gene ontology (GO) enrichment analysis of the *HSP20* gene of in *Citrullus lanatus* revealed found the that *HSP20* gene plays a significant role in various stress responses [21]. For instance, it has been reported that the heterologous expression of the *HSP20* gene of *Triticum aestivum* in *Arabidopsis thaliana* confers resistance to salt and heat stress [22]. Additionally, the *HSP20* family genes of *Arabidopsis thaliana* are upregulated following exposure to drought and salt stress, and have been reported to partake in stress responses [14]. Abiotic stressors such as salt, drought, and low temperatures can induce the expression of the *HSP20* gene of *Saccharum officinarum* [23]. A previous study reported that the *HSP20* gene of *Lycopersicon esculentum* is expressed in large quantities under abiotic and biological stresses [24]. However, the functions of the *HSP20* gene of *C. limonia* remain unclear to date, and especially the specific roles of that *HSP20* in mediating the adaptation of *C. limonia* to adapt the high-calcium environments remain to be investigated to date.

To elucidate the effects of the *HSP20* gene on the growth of *C. limonia* in the karst soils, the *HSP20* genes of in two species of yellow Camellia, namely, the karst species, *C. limonia*, and the non-karst species, *C. nitidissima*, were cloned and were overexpressed heterologously in *Arabidopsis thaliana*. The *HSP20* of Arabidopsis was also overexpressed, and a T-DNA insert mutant was additionally used to determine the functions of the *HSP20* gene under high-calcium stress. Phenotypic observation, energy spectrum scanning, and analysis of the soil physiological indices revealed that the overexpression of *HSP20* gene enhanced the resistance of transgenic *Arabidopsis thaliana* to high-calcium stress, and ClHSP20-OE lines exhibited the highest resistance to high-calcium stress. Additionally, the accumulation of H_2_O_2_ and O_2_^−^ in the leaves of ClHSP20-OE lines was lowest under high calcium stress compared to that of the other plant lines. The palisade tissues in the leaves structure of athspmutant and CnHSP20-OE lines was structurally more tightly arranged than those of the ClHSP20-OE and AtHSP20-OE lines, suggesting that the latter two lines had a superior water holding capacity than the other lines under high-calcium stress. Subsequent transcriptomic analysis revealed that the *HSP20* gene induced the expression of other genes that participated in the high-calcium stress response.

The related genes of ClHSP20-OE lines that were significantly upregulated were involved in various metabolic pathways, including the glycolytic and pentose phosphate pathways; however, these genes were significantly downregulated in CnHSP20-OE lines. In in ClHSP20-OE lines, high-calcium levels induced more differentially expressed genes (DEGs) involved in high-calcium stress, including *FBA5*, *AT5G10770*, and *BBX18*. The expression levels of the *FBA5* and *AT5G10770* genes of ClHSP20-OE lines were significantly higher than those of the CnHSP20-OE lines. However, the expression of *POL*, *AT3G30460*, *DREB1A*, and other genes of AtHSP20-OE lines differed significantly from that of the athspmutant lines. These results highlighted the potential mechanisms underlying the functions of the *HSP20* gene in high calcium environments, and the findings revealed found that the expression of the *HSP20* plays an gene is important role in for the growth of *C. limonia* in the karst regions. The findings obtained herein provide insights for future research on the role and functions of the *HSP20* gene of plants, especially those growing in karst regions. The study also can provides a foundation for the conservation and protection work of endangered yellow Camellia plants.

## Materials and methods

### Vector constructs

**Fig. 1:**
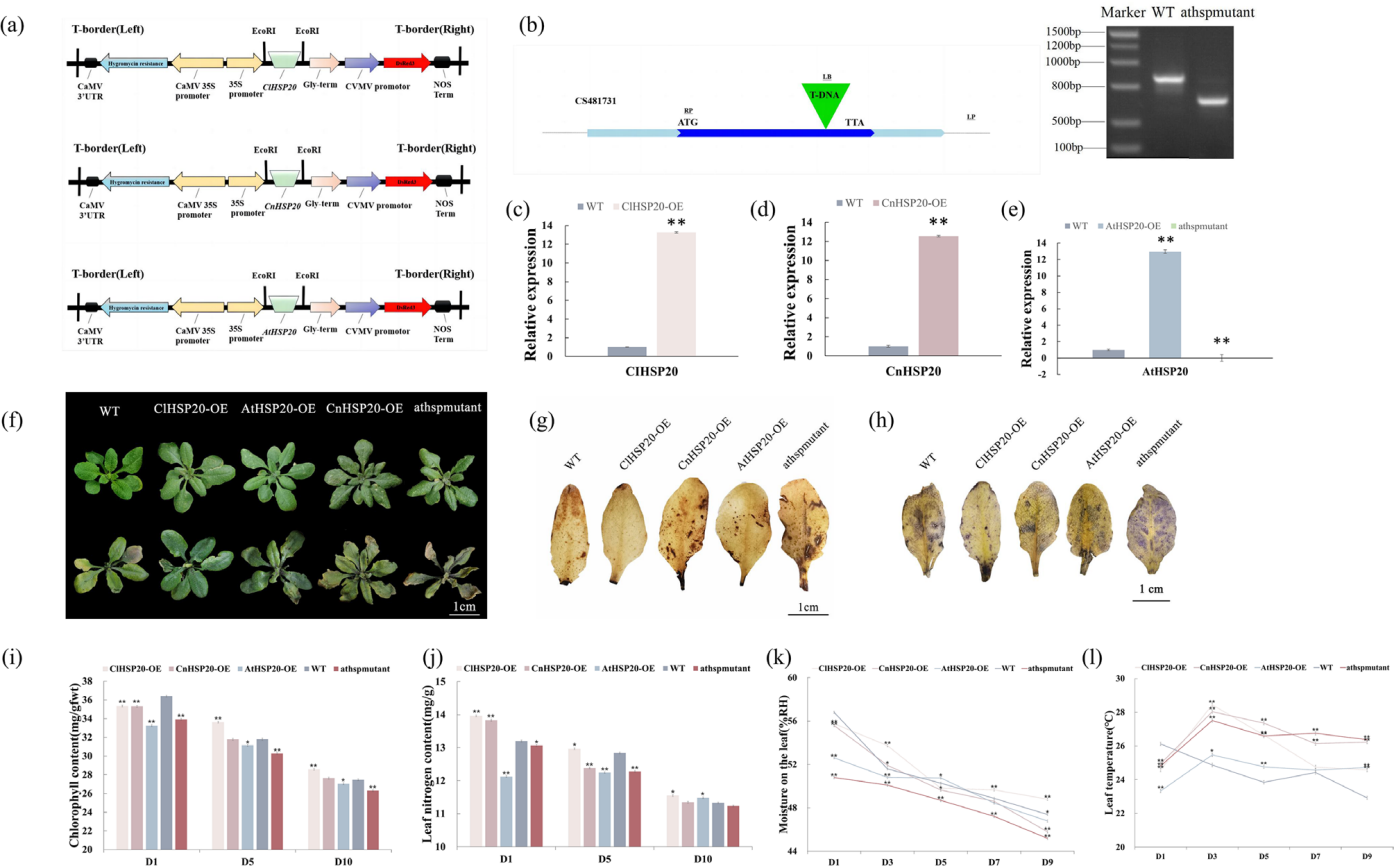
Phenotypes of overexpression of ClHSP20, CnHSP20 and AtHSP20 and the athspmutant lines** (a)**: Schematic diagram of overexpression vector constructed in this study, **(b)**: Diagram of T-DNA insertion mutants used in this study. The full-length gel of PCR confirmation results of athspmutants. The lanes from left to right:1:Marker, 2: WT, 3–5: athspmutant lines, **(c-e)**: Relative expression of *ClHSP20, CnHSP20, AtHSP20* genes in OE and mutant lines. **(f)**: Phenotype of plants after high calcium stress treatment, Bar = 1 cm, **(g)**: DAB staining of WT, OE and athspmutant lines after high calcium stress treatment, Bar = 1 cm, **(h)**: NBT staining of WT, OE and athspmutant lines after high calcium stress treatment, Bar = 1 cm, **(i)**: Chlorophyll content of different lines after high calcium stress treatment (mg/gfwt), **(j)**: Leaf nitrogen content of different lines after high calcium stress treatment (mg/g), **(k)**: Moisture on the leaf of different lines after high calcium stress treatment (%RH), **(l)**: Leaf temperature of different lines after high calcium stress treatment. (*, ** means P value is less than 0.05 and 0.01, respectively)

The *HSP20* genes of *C. limonia* and *C. nitidissima*, denoted as ClHSP20 and CnHSP20, respectively, were cloned in this study [24] (Fig. 1a). According to our previous study [13], different types of yellow Camellia have varying tolerances to high-calcium levels, and gene selection in *C. limonia* is based on the high expression of *HSP20*. The *C. limonia* and *C. nitidissima* plants used in this study were collected and cultivated by our group in the campus of Guangxi Normal University. The homologous *AtHSP20* gene was additionally cloned (AtHSP20) in this study, and the T-DNA insert of the AtHSP20 mutant (No. CS481731) was purchased from the Arabidopsis Biological Resource Center (ABRC; https://abrc.osu.edu). The seeds of the mutant were kindly donated by Bernd Weisshaar. Lisha Zhong and Yuxing Shi planted the seeds and the mutation was confirmed by polymerase chain reaction (PCR) (Fig. 1a and b; Figure S2). The pCAMBIA1303 vector with the 35 S promoter was used for functional overexpression of the *ClHSP20, CnHSP20* and *AtHSP20* genes [25]. The ClHSP20, CnHSP20, and AtHSP20 lines were subsequently transformed using the wild type (WT) as the background, based on the experiments described in earlier studies [26].

### Transformation of Agrobacterium into *Arabidopsis thaliana*

The constructed recombinant plasmid is proposed by plasmid extraction kit, and transferred into GV3101 of Agrobacterium, following which the positive single is identified by colony PCR. The positive bacteria were transferred into WT (Col-0) *Arabidopsis thaliana* using the floral-dip method [27]. The transgenic plants were identified and screened by resistance screening, PCR specific amplification and cloning sequencing [28].

### Selection and planting of *Arabidopsis thaliana* and high-calcium treatment

The transgenic *Arabidopsis thaliana* seeds were selected on the MS medium plates with hygromycin. The positive transgenic seedlings were resistant to hygromycin and were therefore able to grow in the presence of hygromycin. These positive seedlings were evenly moved to a pot containing the configured soil, and placed in a greenhouse at a temperature of 22 ℃, under a light/dark photoperiod of 16 h/8 h, and a relative humidity of 80% [29]. During this period, the growth of the seedlings was observed daily and appropriate quantities of water were added.

The *Arabidopsis thaliana* plants were treated with high calcium after 35 days of germination. The plants in all the experimental groups were treated with 200 mmol/L CaCl_2_ and kept under a light/dark photoperiod of 16 h/8 h at a relative humidity of 80%. This treatment lasted for 8–10 days at 22 ℃, following which the plants were treated with 300 mmol/L CaCl_2_ for 2–4 days [13].

### Collection of data regarding the growth environment of the experimental groups

The physiological and biochemical indices of the plants and soils were determined using an RS485 soil temperature and humidity sensor and a JXBS-3001-PH soil pH sensor [13]. The physiological and biochemical indices of the soil included soil temperature and humidity, pH, and contents of soil nitrogen, phosphorus, and potassium. The physiological and biochemical indices of the leaves, including the contents of chlorophyll and nitrogen in the leaves, leaf surface temperature, and moisture content, were determined using a TYS-4 N chlorophyll analyzer [30]. The photosynthetic parameters of the leaves of *Arabidopsis thaliana*, including the carbon dioxide conductance, transpiration rate, intercellular carbon dioxide concentration, stomatal conductance, total conductance, and other parameters, were measured using an LI-6800 photosynthesizer [31].

### Staining and phenotypic observation of *Arabidopsis thaliana*

For staining, 3,3-N-diaminobenzidine tetrahydrochloride (DAB) and nitrotetrazolium blue chloride (NBT) were purchased from Phygene Company, and staining was performed according to the method described by Liu et al [32]. Sample fixation and preparation by paraffin sectioning [33] and and scanning electron microscopy [34] were performed as previously described.

### Preparation of transcriptome material and RT-qPCR analysis

The ClHSP20 and CnHSP20 transgenic plants with heterozygous *HSP20* overexpression, AtHSP20 transgenic plants with homozygous *HSP20* overexpression, WT, and athspmutant plants were used as the experimental materials. Three biological replicates were used for each transgenic line, and three biological replicates were used as control for the WT, resulting in a total of fifteen samples. The leaves of the WT, ClHSP20-OE, CnHSP20-OE, AtHSP20-OE, and athspmutant Arabidopsis plants were collected after 20 days of treatment with high calcium and stored in nitrogen solution, and subsequently transferred from liquid nitrogen to an ultra-low temperature freezer at -80 °C [6]. The reaction materials for qRT-PCR were designed using the Primer 5 software (Supplementary Figure S1). The qPCR mixture consisted of 1 µl of each of the upstream and downstream primers, 25 µl of PCR buffer, and 10 µl of SYBR Green Mix reagent purchased from Takara Bio, and the total volume of the PCR was adjusted to 50 µl by adding ddH_2_O. The conditions of the PCR cycle were as follows: initial denaturation at 94 ℃ for 2 min followed by denaturation at 98 ℃ for 10 s, annealing at 58 ℃ for 30 s, 32 cycles of extension at 68 ℃ for 30 s, and a final extension at 68 °C for 2 min. The melting curves were generated at the third step for estimating reaction specificity [24] (Fig. 1c, d and e).

### Transcriptome sequencing, and library construction and library inspection

The concentration and purity of the RNA were assessed using a NanoDrop2000 spectrophotometer. The integrity of the RNA was determined by agarose gel electrophoresis, and the RIN value was determined using an Agilent 2100 Bioanalyzer system. Qualified total RNA extracted from tissue samples was used for library construction using the Illumina TruseqTM RNA sample prep Kit method based on the Illumina Noveseq 6000 sequencing platform [13]. PE150 was used for sequencing. High-throughput sequencing was performed using a certain concentration of libraries collected after inspecting the qualified library.

### Analysis and assembly of transcriptome sequencing data

The quality control data were compared with the reference genome to obtain the mapped data for subsequent analysis, and the quality of the results of sequence comparison was evaluated. The mapped reads were assembled and spliced using Cufflinks [35] or StringTie [36] software based on existing reference genes for subsequent analysis.

### Gene function annotation

The assembled gene sequences of the ClHSP20, CnHSP20, and AtHSP20 transgenic lines and WT and mutants were matched against eight databases, namely, GO, KEGG, COG, NR, Swiss-Prot, Pfam, Total anno, and Total [13].

### Gene expression and analysis of differential expression

Based on the quantitative expression data, the genes that were differentially expressed between the two groups were identified by differential gene expression analysis. Using DEseq2 from the analysis of the genes differentially expressed the read count data identified genetic variations in the summary, screening of threshold value is: |log_2_FC|>=1&padjust < 0.05. Subsequently, The primary functions and metabolic pathways of the screened differentially expressed genes were subsequently determined by KEGG pathway and GO functional enrichment analyses [37, 38].

### RT-qPCR for the marker genes and validation of transcriptome

The gene-specific primers were designed using the qPCR Primer Database [39]. A housekeeping gene (ACTIN) that is constitutively expressed in Arabidopsis was used as a reference for normalization and analyzed using three biological replicates for the qPCR analysis. RNA isolation and RT-qPCR analysis were performed according to the methods described in a previous study [24]. The abiotic stress marker genes were also verified by qPCR. A total of 15 DEGs and genes were randomly selected for RT-qPCR analysis for validating the transcriptome data. As depicted in Supplementary Figure S3, the results of RT-qPCR were generally consistent with the results of transcriptome analysis.

## Results

### *HSP20* genes suppressed the reduction in the contents of leaf chlorophyll and nitrogen, reduced the leaf moisture content, and improved the free radical scavenging ability under high calcium stress

To study the effect of *HSP20* genes in plants, the *ClHSP20* genes, *CnHSP20* genes, and *AtHSP20* genes were amplified by PCR and constructed into plant expression vectors. The WT lines were transformed via the Agrobacterium inflorescence infection method to obtain the ClHSP20-OE, CnHSP20-OE, and AtHSP20-OE lines, and the athspmutant was obtained by knockout of *AtHSP20* genes. The coding regions of the *ClHSP20*, *CnHSP20* and *AtHSP20* (*AT1G07400*) genes were 519,639 and 471 bp long, respectively(Supplementary Figure S1). and the differences in gene function could be attributed to differences in gene structure.

The WT and athspmutant lines and the transgenic Arabidopsis plants overexpressing *ClHSP20*, *CnHSP20* and *AtHSP20 genes* were treated with high calcium. The results demonstrated that the ClHSP20-OE lines exhibited the strongest resistance to high-calcium stress following 20 days of treatment with high calcium, and their growth status was superior to that of the other lines and the leaves appeared to shrink. Followed by WT, AtHSP20-OE, CnHSP20-OE and athspmutants lines had the worse growth status and poor resistance to high calcium stress (Fig. 1f). The abiotic stress marker genes were verified by qPCR. The findings revealed that compared with WT line, the expressions of the abiotic stress marker genes was relatively higher in ClHSP20-OE, CnHSP20-OE and AtHSP20-OE lines, but low in athspmutant lines (Supplementary Figure S4). The results of DAB staining revealed that the leaves of the WT, CnHSP20-OE and athspmutant lines were deeply stained and the accumulation of H_2_O_2_ was high in the leaves (Fig. 1g). The results of NBT staining revealed that the staining intensity of the leaves of ClHSP20-OE lines was relatively low and the accumulation of superoxide anion in the leaves was lowest among all the plant lines; however, the accumulation of superoxide anion was highest in the leaves of CnHSP20-OE lines (Fig. 1h). These findings suggested that the calcium resistance of ClHSP20-OE lines was superior to that of other OE lines under high-calcium stress, and the calcium resistance of athspmutant lines was the weakest, which indicated that *HSP20* genes can improve resistance to high-calcium stress.

The contents of chlorophyll and nitrogen in the leaves were highest on the second day of high-calcium treatment. The contents of leaf chlorophyll and nitrogen decreased gradually with an increase in the duration of treatment; however, the reduction was lowest in ClHSP20-OE and AtHSP20-OE lines, and most pronounced in athspmutant lines. The reduction in the contents of leaf chlorophyll and nitrogen in CnHSP20-OE lines ranged between that of the ClHSP20-OE and athspmutant lines. The contents of chlorophyll and nitrogen in the leaves of ClHSP20-OE lines were higher than those of CnHSP20-OE, AtHSP20-OE, WT and athspmutant lines. However, the contents of chlorophyll and nitrogen in the leaves of athspmutant lines were lower than those of WT lines. The chlorophyll content, leaf nitrogen content of CnHSP20-OE and AtHSP20-OE lines were between ClHSP20-OE and athspmutant lines. Compared to that of the WT lines, the chlorophyll content in the leaves of ClHSP20-OE, CnHSP20-OE, AtHSP20-OE, and athspmutant lines decreased by 66.7%, 77.8%, 66.7%, and 88.9%, respectively, under high-calcium stress (Fig. 1i). Additionally, high-calcium stress decreased the nitrogen content in the leaves of ClHSP20-OE, CnHSP20-OE, AtHSP20-OE, and athspmutant lines decreased by 50%, 100%, 50%, and 150%, respectively(Fig. 1j). The moisture content of the leaves of ClHSP20-OE, CnHSP20-OE, AtHSP20-OE, and athspmutant lines decreased by 100%, 70%, 100% and 60%, respectively, compared to that of the WT lines (Fig. 1k and l). These results suggested that the *HSP20* gene can suppress the reduction in the contents of chlorophyll and nitrogen in the leaves under high-calcium stress, and reduce the moisture content in the leaves. The reduction in foliar moisture content was primarily attributed to the reduction in the rate of transpiration following treatment with high calcium, suggesting that this may be one of the results of adaptation to high calcium environment, and *ClHSP20* gene could play the most obvious role. consequently, the resistance of ClHSP20-OE lines to high-calcium stress was most superior, whereas the resistance of the athspmutant lines was most inferior, and the resistance of CnHSP20-OE and AtHSP20-OE lines ranged between that of the ClHSP20-OE and athspmutant lines.

### *HSP20* gene contribute to *C. limonia* to adapt to high calcium environment by altering stomatal aperture and leaf calcium content

**Fig. 2:**
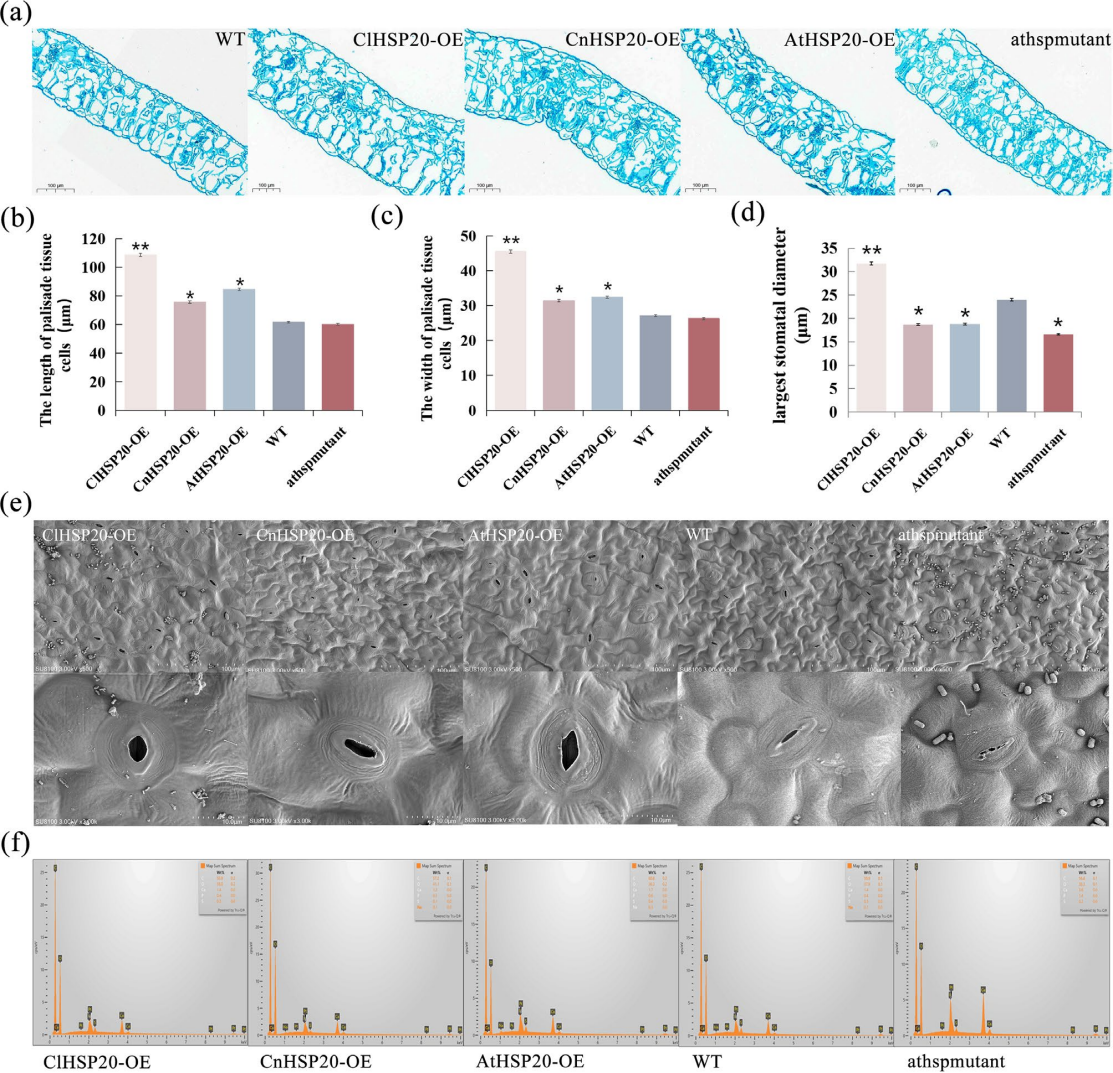
Paraffin sections and scanning electron microscopy of leaves under high calcium stress** (a)**: Leaves sections of WT, ClHSP20-OE, AtHSP20-OE, CnHSP20-OE and athspmutant under high calcium treatment, Bar = 100 μm, **(b)**: The length of palisade tissue cells under high calcium treatment (µm), **(c)**: The wide of palisade tissue cells under high calcium treatment(µm), **(d)**: The largest stomatal diameter of the stoma of each group under high calcium stress, **(e)**: Electron microscope scanning (SEM) images of the leaves of ClHSP20-OE, CnHSP20-OE, AtHSP20-OE, WT and athspmutant lines treated with high calcium, **(f)**: The combination of energy spectrum graphs of all the lines. (*, ** means P value is less than 0.05 and 0.01, respectively)

To determine the alterations in the cellular microstructure of the foliar tissues, paraffin sections of the leaf tissues were prepared and observed (Fig. 2a). The findings revealed that the average length and width of the cells in the fenestrated tissues of ClHSP20-OE lines were 109 μm and 46 μm, respectively, after 20 days of high-calcium stress, whereas those of the athspmutant lines were 60 μm and 26 μm, respectively. The average length and width of the cells in the palisade tissues of CnHSP20-OE and AtHSP20-OE lines ranged between those of ClHSP20-OE and AtHSP20-OE lines. Compared to those of the WT lines, the average length and width of the cells in the palisade tissues of ClHSP20-OE lines increased significantly by 76.1% and 67.9%, respectively(Fig. 2b and c). Therefore, the structure of the palisade tissues of athspmutant lines was more compact, and the cellular water content was lower than those of ClHSP20-OE, CnHSP20-OE, and AtHSP20-OE lines. The density of the spongy tissues of ClHSP20-OE lines was the highest among all the plant lines tested herein, and there were 9 cells per 0.01 mm^2^ of spongy leaf tissue. These results suggested that the *ClHSP20* gene may enhance water retention in lines under high-calcium stress by increasing the cellular packing density in spongy tissues and inducing cellular enlargement in foliar palisade tissues.

The leaves were further examined by scanning electron microscopy for studying the structural alterations in the stomata in the mesophyll tissues of leaves(Fig. 2e). The results of scanning electron microscopy revealed that the diameter of the stomatal apertures of ClHSP20-OE lines was the largest, being 31.75 μm on average, whereas that of the athspmutant lines was smallest, being only 16.6 μm on average (Fig. 2d). The dimension of the stomatal aperture may affect photosynthetic efficiency and the transpiration rate of plants. The stomatal aperture of ClHSP20-OE lines was the most optimum, and these lines were therefore likely to exhibit optimal photosynthetic efficiency for adapting to the high-calcium environment. The elements on the laminar surface, including carbon, oxygen, sodium, phosphorus, sulfur, and calcium, were scanned by analyzing the leaf energy spectrum. Of these elements, the differences in calcium content was most distinct among the line groups. The percentage of calcium in the leaves of the ClHSP20-OE, CnHSP20-OE, AtHSP20-OE, WT, and athspmutant lines was 1.37%, 1.21%, 1.72%, 1.38%, and 3.56%, respectively, which revealed that the percentage of calcium was relatively low in ClHSP20-OE lines. This implied that these lines had made optimum use of the extra calcium and had adopted different mechanisms for removing the Ca^2+^ ions via their physiological processes to withstand the high-calcium stress. However, the percentage of calcium in the leaves of athspmutant lines was the highest, indicating a weaker ability to eliminate the extra calcium, which led to the accumulation of calcium in the leaves. These findings suggested that athspmutant lines were incapable of resisting the high-calcium stress (Fig. 2f; Table 1).

**Table 1:** Leaf content percentages (WT% sigma) of elements detected by energy spectrum in all lines

Elements	ClHSP20-OE lines	CnHSP20-OE lines	AtHSP20-OE lines	WT	athspmutant lines
C	59.93(0.17)	57.20(0.11)	60.75(0.15)	59.85(0.14)	56.55(0.14)
O	38.04(0.17)	41.07(0.11)	36.30(0.16)	37.94(0.14)	38.28(0.14)
Na		0.09(0.01)	0.26(0.02)	0.11(0.01)	
P	0.37(0.01)	0.31(0.01)	0.61(0.01)	0.40(0.01)	1.38(0.02)
S	0.28(0.01)	0.12(0.01)	0.35(0.01)	0.33(0.01)	0.22(0.01)
Ca	1.37(0.02)	1.21(0.01)	1.72(0.02)	1.38(0.01)	3.56(0.02)
Total	100%	100%	100%	100%	100%

### Heterologous expression of *HSP20* gene of different *Camellia* species in high calcium resistance in *Arabidopsis thaliana* alter photosynthetic rate of plants

**Fig. 3:**
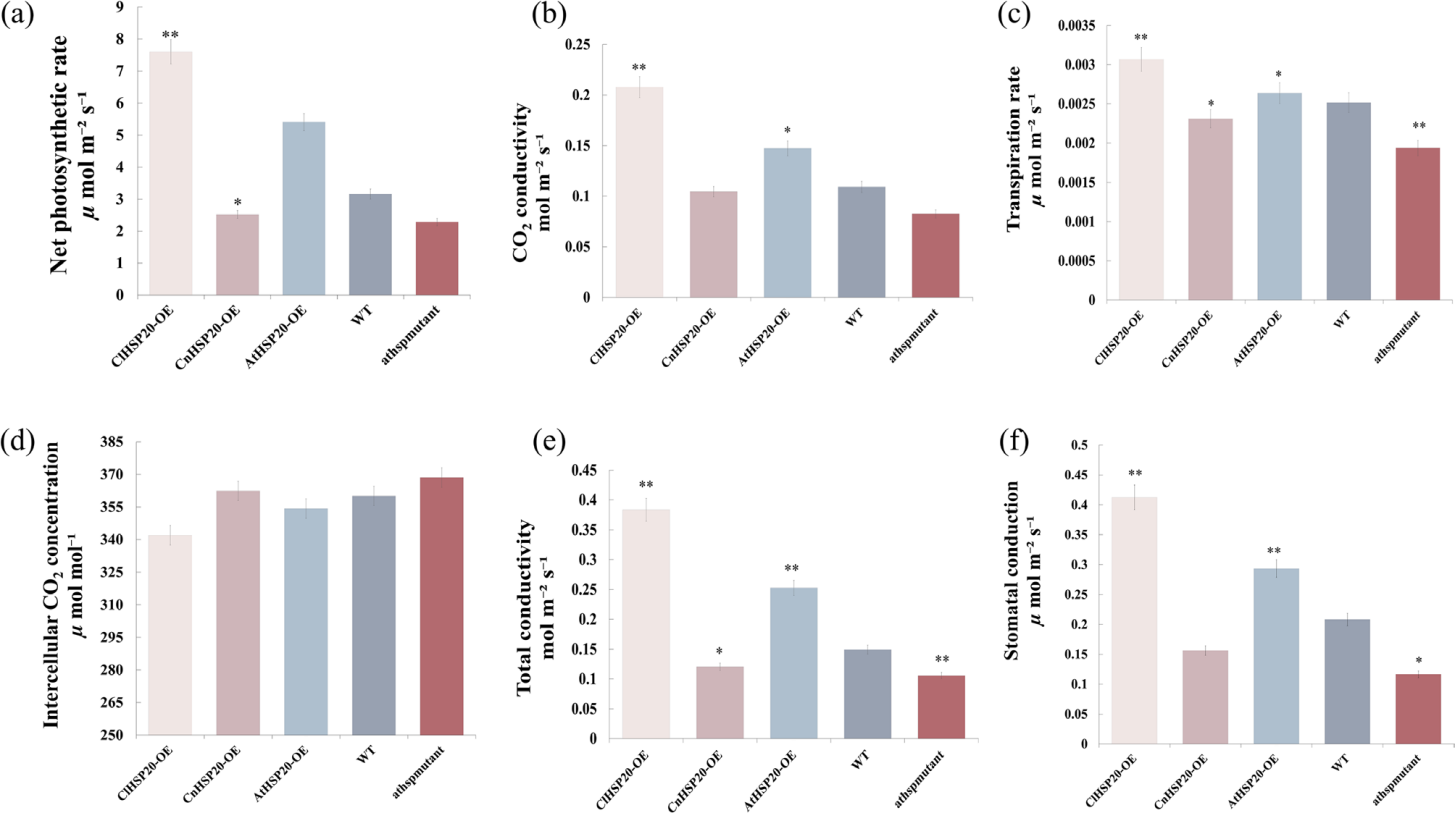
Photosynthetic system analysis of wild-type and transgenic Arabidopsis Thaliana with overexpression of different *HSP20* genes under high calcium stress** (a)**: Net photosynthetic rate (µ mol m^− 2^^− 1^), **(b)**: CO_2_ conductivity (mol m^− 2^^− 1^), **(c)**: Transpiration rate (µ mol m^− 2^^− 1^), **(d)**: Intercellular CO_2_ concentration (µ mol mol^− 1^), **(e)**: total conductivity (mol m^− 2^^− 1^), **(f)**: stomatal conductance (µ mol m^− 2^^− 1^). (*, ** means *P* value is less than 0.05 and 0.01, respectively)

Analysis of the chlorophyll contents and diameters of the stomatal apertures revealed that the physiological phenotypes of the ClHSP20-OE, AtHSP20-OE, CnHSP20-OE, athspmutant, and WT lines altered under high-calcium stress, indicating that *HSP20* may affect the photosynthetic system of *Arabidopsis thaliana.* To further investigate the effect of *HSP20* on the photosynthetic system, the photosynthetic systems of ClHSP20-OE, CnHSP20-OE, AtHSP20-OE, and athspmutant lines were analyzed, using the WT lines as the control. The results demonstrated that the net photosynthetic rate of ClHSP20-OE lines was higher and that of the athspmutant lines was lower than that of the WT lines under high-calcium stress (Fig. 3a). Compared with WT lines, the net photosynthetic rate, carbon dioxide conductance, transpiration rate, total conductance, and stomatal conductance of ClHSP20-OE lines were highest, whereas athspmutant lines were the lowest. The net photosynthetic rate, carbon dioxide conductance, transpiration rate, total conductance, and stomatal conductance of the CnHSP20-OE and AtHSP20-OE lines ranged between those of ClHSP20-OE and athspmutant lines. The carbon dioxide conductivity of ClHSP20-OE and CnHSP20-OE plants was 0.21 mol m^−^² s^−^¹ and 0.10 mol m^−^² s^−^¹, respectively (Fig. 3b). The transpiration rates of ClHSP20-OE and AtHSP20-OE lines were higher than those of WT lines under high-calcium stress, and the transpiration rate of the WT lines was higher than those of CnHSP20-OE and athspmutant lines (Fig. 3c). Analysis of the photosynthetic data revealed that the transpiration rate of ClHSP20-OE lines was the highest, whereas that of the athspmutant lines was lowest, suggesting that the *HSP20* gene may have affected the transpiration rate. The intercellular carbon dioxide concentration of ClHSP20-OE lines was the lowest among all the plant lines, whereas that of the athspmutant lines was highest. The intercellular carbon dioxide concentration of the WT lines was higher than that of ClHSP20-OE lines but lower than that of athspmutant lines (Fig. 3d). Compared with WT lines, the total conductivity of the ClHSP20-OE lines was the highest and that of the athspmutant lines was the lowest. The total conductivity of the ClHSP20-OE and AtHSP20-OE lines was higher than that of the WT by 156.8% and 69.2%, respectively. However, the total conductivity of athspmutant and CnHSP20-OE lines was lower than that of the WT by 29.2% and 19.3%, respectively (Fig. 3e). The stomatal conduction of the ClHSP20-OE and AtHSP20-OE lines was higher that of the WT lines by 98.2% and 41.0%, respectively. However, the stomatal conduction of the CnHSP20-OE and athspmutant lines was lower than that of the WT by 24.9% and 44.0%, respectively (Fig. 3f). These findings indicated that the stomatal conductance of ClHSP20-OE lines was consistently higher than that of CnHSP20-OE lines. These results suggested that the *HSP20* gene clearly increased the transpiration rate by increasing stomatal conductance and reducing the intercellular carbon dioxide concentration, which could ultimately increase the photosynthetic rate.

### *HSP20* gene can slow down the decrease of phosphorus, kalium, and nitrogen contents in soil and increase soil humidity

**Fig. 4:**
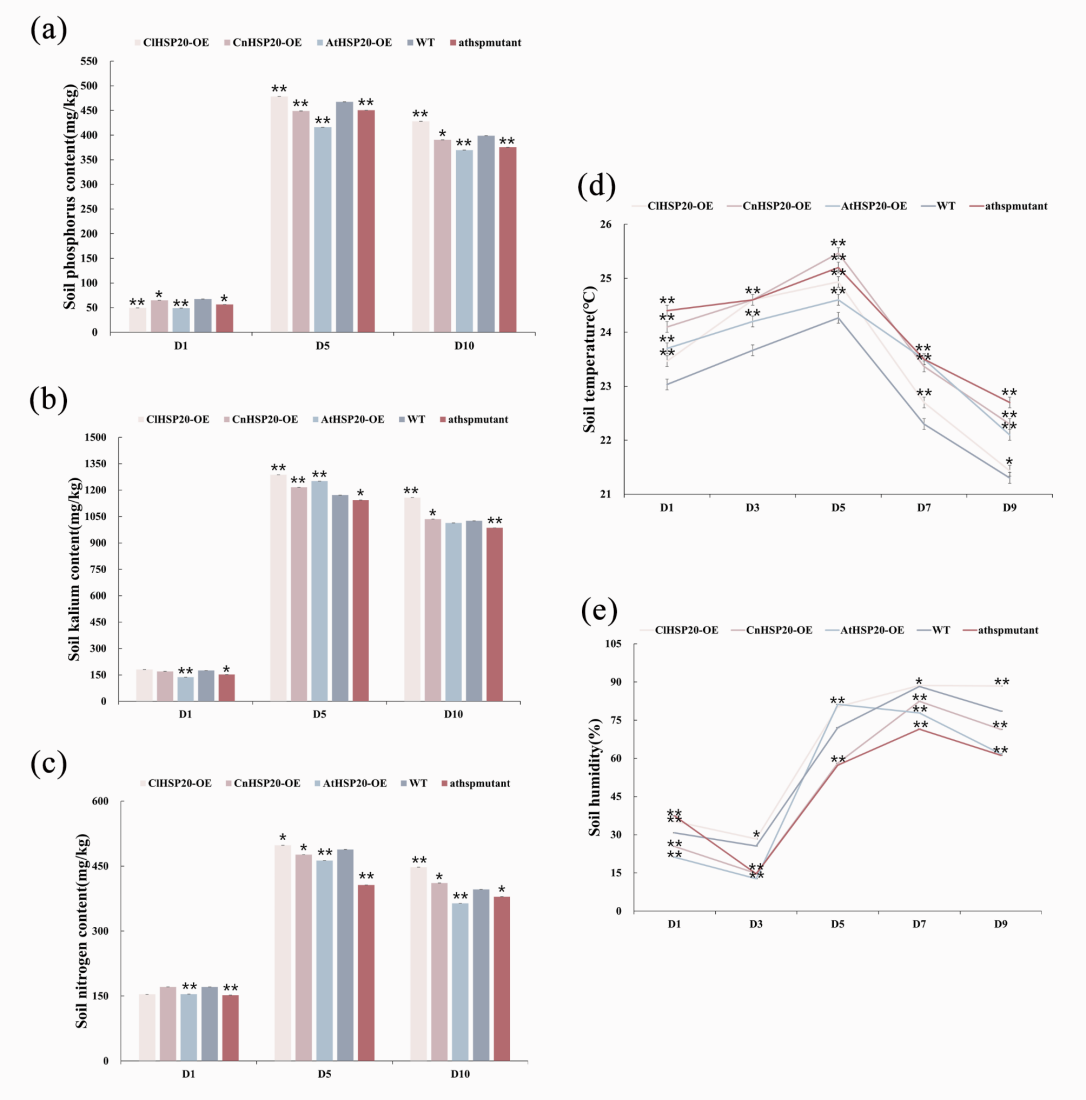
Soil physiological indexes of WT and ClHSP20-OE, CnHSP20-OE, AtHSP20-OE, and athspmutant under high calcium stress** (a)**: Soil phosphorus content(mg/g), **(b)**: Soil kalium content(mg/g), **(c)**: Soil nitrogen content(mg/g), **(d)**: Soil temperature (℃), **(e)**: Soil humidity (%). (*, ** means P values less than 0.05 and 0.01, respectively)

To understand the effect of the *HSP20* genes of plants on the soil environment under high-calcium stress, the contents of soil phosphorus, kalium, and nitrogen as well as the soil temperature, soil humidity, and physical data were determined and analyzed following treatment with high of calcium. The findings revealed that the contents of soil phosphorus, kalium, and nitrogen were highest on the fifth day of treatment. However, the contents of soil phosphorus, kalium, and nitrogen decreased gradually with an increase in the duration of treatment, and the reduction was smallest in ClHSP20-OE lines and largest in athspmutant lines. The reduction in the CnHSP20-OE and AtHSP20-OE lines ranged between those of ClHSP20-OE and athspmutant lines. Compared to those of the WT, the contents of soil phosphorus, kalium, and nitrogen were highest for ClHSP20-OE lines and lowest for athspmutant lines, whereas those of CnHSP20-OE and AtHSP20-OE lines ranged between those of the ClHSP20-OE and athspmutant lines. The soil phosphorus contents for the ClHSP20-OE, CnHSP20-OE, AtHSP20-OE, and athspmutant lines were lower than that of the WT by 66.7%, 84%,73.9%, and 109%, respectively, under high-calcium stress (Fig. 4a). The soil kalium contents for the ClHSP20-OE, CnHSP20-OE, AtHSP20-OE, and athspmutant lines were lower than that of the WT by 88.4%, 123.2%, 108.3%, and 162.3%, respectively (Fig. 4b). Similarly, the soil nitrogen contents for the ClHSP20-OE, CnHSP20-OE, AtHSP20-OE, and athspmutant lines were lower than that of the WT by 29.3%, 71.7%, 55.4%, and 95%, respectively (Fig. 4c). The soil pH did not undergo any alterations and remained constant at 7.0 for all the plant groups during the entire process of treatment. The alterations in soil temperature did not exhibit any obvious trends for all the plant groups (Fig. 4d). The soil humidity for ClHSP20-OE, CnHSP20-OE, AtHSP20-OE, and athspmutant lines was higher than that of the WT by 110.4%, 95.8%, 85.4%, and 50%, respectively (Fig. 4e). These results suggested that the *HSP20* gene can suppress the reduction in the contents of soil phosphorus,kalium, and nitrogen and increase soil humidity under high-calcium stress. The variations in the contents of soil phosphorus, kalium, and nitrogen reflect the stability of phosphorus, kalium, and nitrogen utilization efficiency, and the findings revealed that the *ClHSP20* gene might play a prominent role in stabilizing the nutrient utilization efficiency. Therefore, these results indicated that the phosphorus, kalium, and nitrogen utilization efficiency of ClHSP20-OE lines is more stable, which can be conducive to the survival of these lines under high-calcium stress. The survivability potential of athspmutant lines was the most inferior among all the plant lines, and that of the CnHSP20-OE and AtHSP20-OE lines ranged between those of ClHSP20-OE and athspmutant lines.

### Transcriptome analyses of ClHSP20-OE and CnHSP20-OE lines reveal *HSP20* gene function in karst adaptation of yellow Camellia

**Fig. 5:**
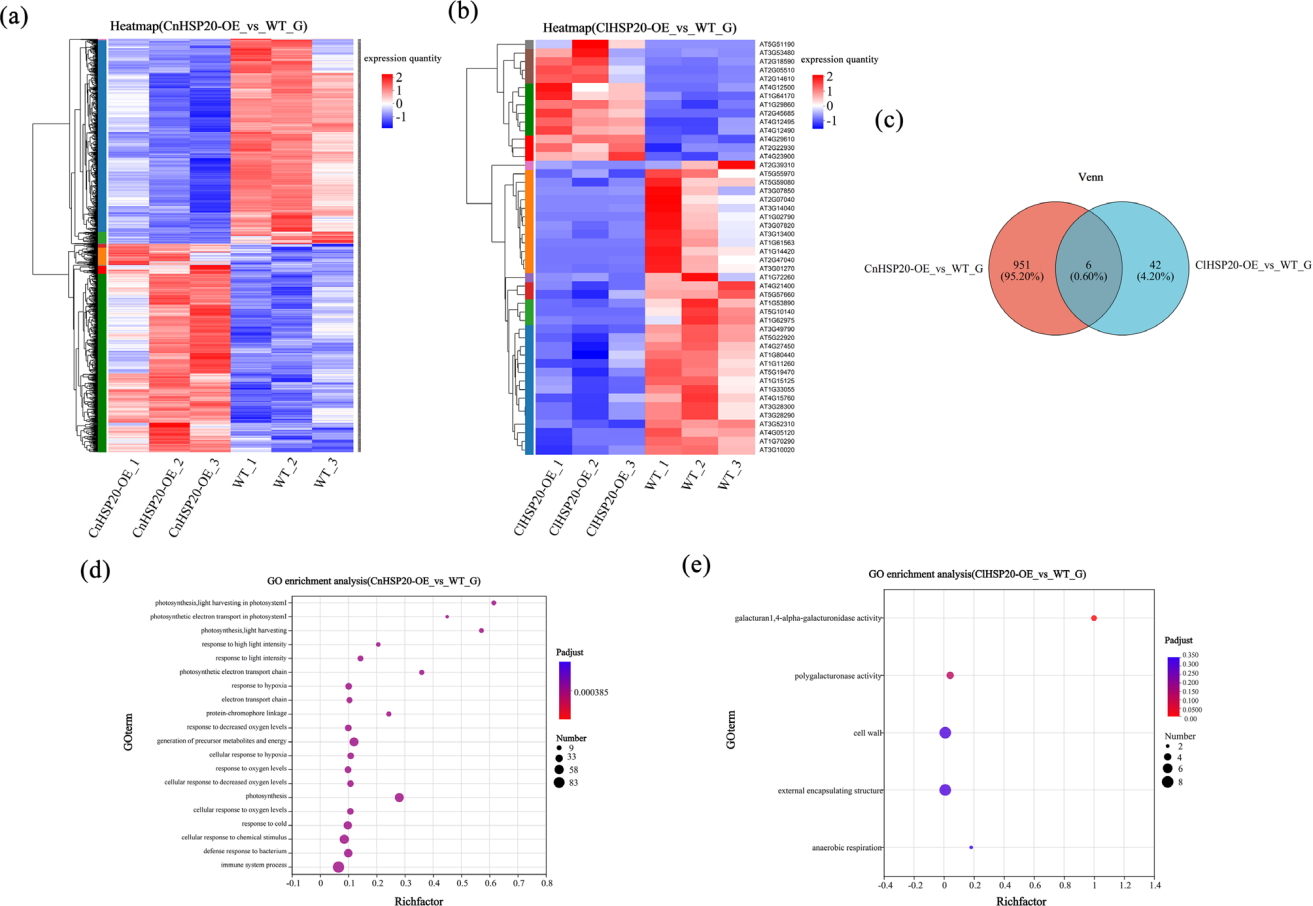
Transcriptomic analysis of ClHSP20-OE and CnHSP20-OE plants differentially expressing HSP20 under high calcium stress** (a **and** b)**: heat maps of ClHSP20-OE and CnHSP20-OE lines differentially expressing *HSP20* under high calcium stress (The numbers against each plant line (e.g. ClHSP20-OE_1, ClHSP20-OE_2, ClHSP20-OE_3) indicate three biological replicates. Red represents higher expression of the gene in this sample, and blue represents lower expression.), **(c)**: Venn diagram of ClHSP20-OE and CnHSP20-OE lines (*P* ≤ 0.05, error < 0.05), **(d **and** e)**: GO enrichment bubble map of ClHSP20-OE and CnHSP20-OE lines differentially expressing *HSP20* gene under high calcium stress

To determine the potential mechanism underlying the effect of the *HSP20* gene in the karst adaptation of yellow camellia, the ClHSP20-OE, CnHSP20-OE, and WT lines were treated with high calcium, and leaf samples were collected for transcriptome analyses. The WT was used as the control for identifying the gene sets that were activated in response to high-calcium stress. Heatmaps were subsequently generated by hierarchical clustering, which revealed that the genes clustered into two groups(Fig. 5a and b). In CnHSP20-OE and WT lines and in ClHSP20-OE and WT lines, there were significant differences in gene expression levels and gene expression patterns, indicating that there were differences in the expression patterns of *ClHSP20* and *CnHSP20* and *AtHSP20* genes. A total of 48 and 957 DEGs were detected in ClHSP20-OE and CnHSP20-OE lines, respectively, by comparing their gene expression levels to those of the WT(Figs. 5c). Further analysis showed that there were 6 common DEGs in ClHSP20-OE vs. WT and CnHSP20-OE vs. WT (include *PR1, AT2G05510, AT1G15125, ABCG37, WRKY71, THI2.1*).

**Fig. 6:**
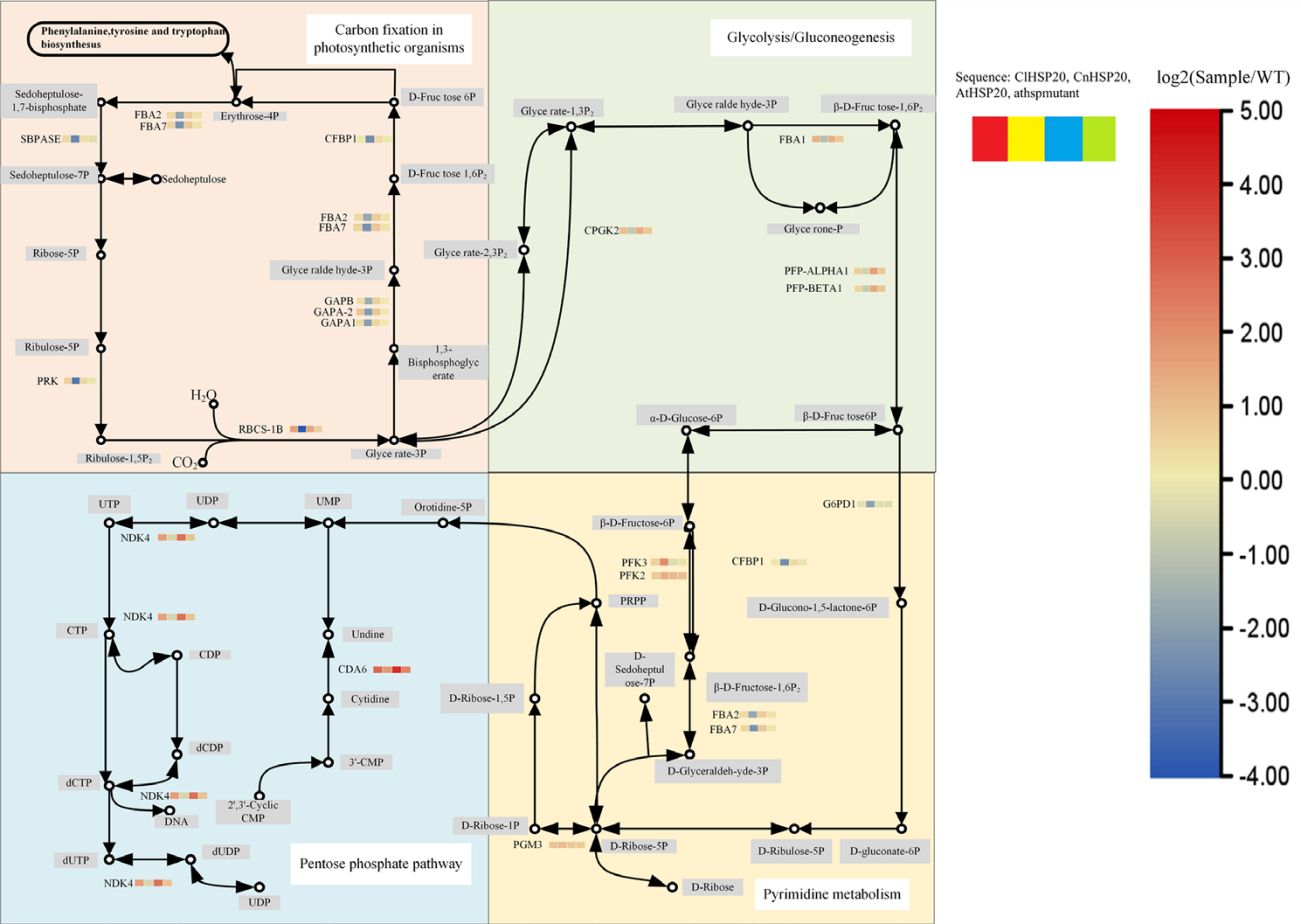
The relative pathways which key differential expressed genes were enriched. All the pathways were drawn according to the to the analysis result from our transcriptome data. KEGG database was used to search and screen the different expressed genes and the pathways that enriched most key different expressed genes was selected and shown in this Fig. [38]. KEGG also permit us to use the maps information in this figure

Heatmap clustering analysis was performed for the ClHSP20-OE and CnHSP20-OE lines for determining the biological processes that the DEGs are involved in(Fig. 5a and b), and enrichment function of gene ontology (GO) using seqenrichment (Fig. 5d and e). The findings revealed that DNA replication and cellular proliferation were significantly upregulated in ClHSP20-OE and CnHSP20-OE lines under high-calcium stress, and were especially pronounced in ClHSP20-OE lines, indicating that their cell division and differentiation capacity were superior to those of CnHSP20-OE lines under high-calcium stress. Through GO enrichment analysis, it was found that different degrees of gene enrichment were produced in each experimental group in response to high calcium environment, and a large number of genes were expressed to resist high calcium stress. The CnHSP20-OE lines had the highest number of genes associated with immune system processes, photosynthesis, and precursor metabolites and energy production compared to WT. The genes were mostly enriched in photosynthesis and light harvesting system processes, and the results of enrichment analysis differed significantly from those of the WT(Fig. 5d). Compared with WT, the number of genes enriched in cell wall and external encapsulating structure terms was highest in ClHSP20-OE lines, and the degree of enrichment of the genes related to galacturan 1, 4-alpha-galacturonidase was highest in these lines(Fig. 5e). In order to further identify potential control networks, DEGs enrichment analysis was performed between gene sets, and relevant KEGG pathway maps were obtained (Fig. 6). KEGG enrichment analysis revealed that photosynthetic carbon fixation and chlorophyll metabolism were upregulated in ClHSP20-OE lines and downregulated in CnHSP20-OE lines. The related genes of ClHSP20-OE lines were significantly up-regulated in biological metabolic pathways such as glycolysis pathway and pentose phosphate, while those of CnHSP20-OE lines were significantly down-regulated. This finding suggested that ClHSP20-OE lines could have superior photosynthetic capacity and biological metabolism under high-calcium stress. Analysis of the KEGG pathway map and previous phenotypic analyses revealed that the majority of genes regulating photosynthetic efficiency was downregulated in all the groups under high-calcium stress; however, the rate of downregulation was least in ClHSP20-OE lines. Through DEGs and KEGG results, it is found that compared with CnHSP20-OE lines, *FBA5* gene was up-regulated 9.1fold change in ClHSP20-OE lines. The *FBA5* gene not only regulates the photosynthetic rate, but also plays a regulatory role in pyrimidine metabolism. The findings indicated that the overexpression of *HSP20* regulated multiple pathways via *FBA5* and other genes with multiple regulatory functions, for resisting the high-calcium stress in a combined manner. The *AT5G10770* gene encodes aspartic proteases (APs), and the *AT5G10770* gene of ClHSP20-OE lines was upregulated by 2.6-fold compared with CnHSP20-OE lines. APs are mainly distributed in guard cells, and the up-regulation of APs genes is conducive to controlling stomatal opening and closing, and photosynthesis in plants [40], which enables plants to adjust to the stressor. Most other genes in all pathway were up-regulated 1 ~ 3fold change in ClHSP20-OE lines and down-regulated 1 ~ 4fold change in CnHSP20-OE lines (Fig. 6). Altogether, these findings suggested that the *HSP20* gene maintained the photosynthetic rate and normal metabolism by regulating the expression of key genes in *C. limonia* under high-calcium stress, and jointly resist the effect of high calcium stress.

### Transcriptome analysis of AtHSP20-OE and athspmutant was conducted to explore the roles of *HSP20* gene in *Arabidopsis thaliana* under high calcium stress

**Fig. 7:**
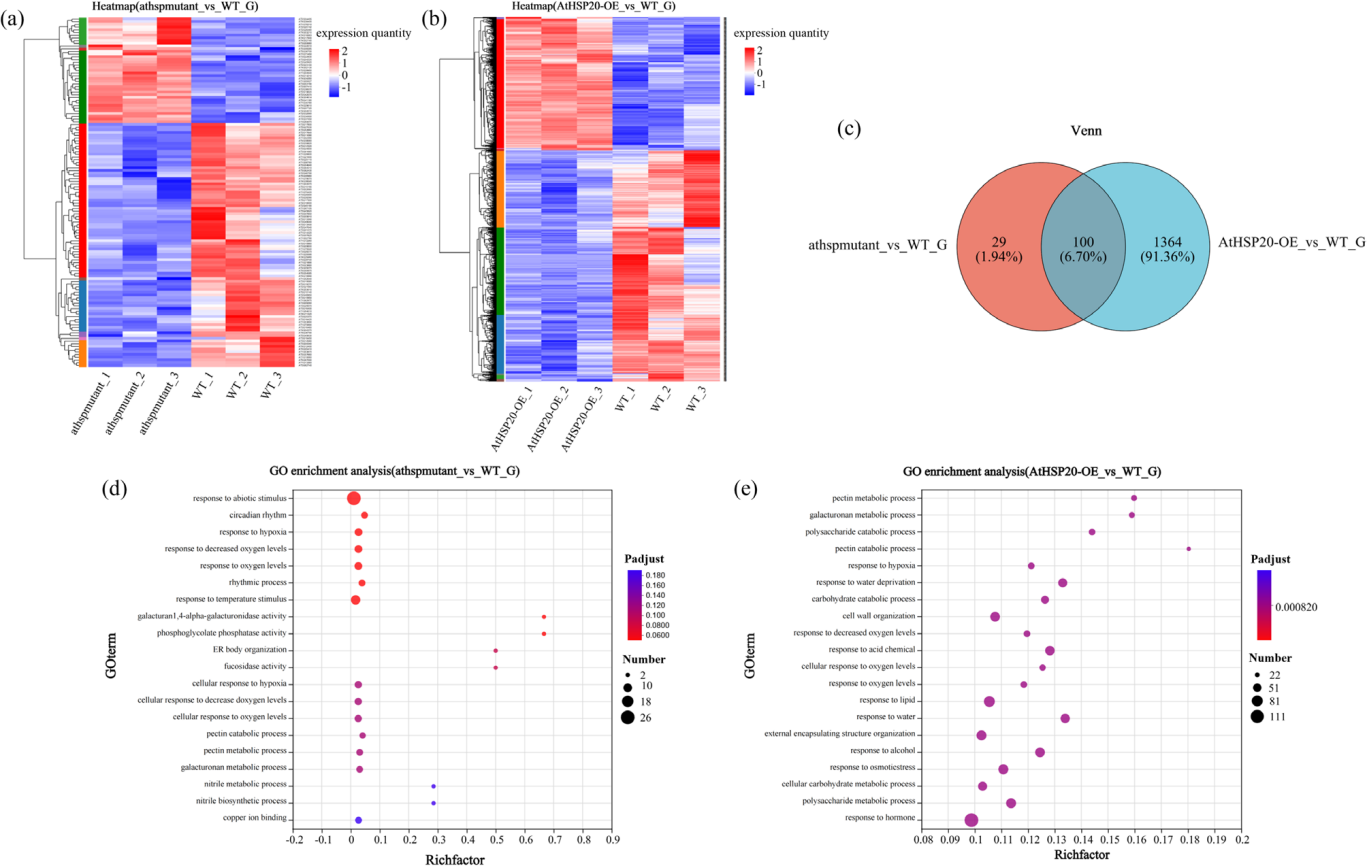
Transcriptomic analysis of AtHSP20-OE and athspmutant lines differentially expressing *HSP20* gene under high calcium stress** (a **and** b)**: Aggregation heat maps of AtHSP20-OE and athspmutant lines differentially expressing *HSP20* gene under high calcium stress (The numbers against each plant line (e.g. ClHSP20-OE_1, ClHSP20-OE_2, ClHSP20-OE_3) indicate three biological replicates. Red represents higher expression of the gene in this sample, and blue represents lower expression.), **(c)**: Venn diagram of AtHSP20-OE and athspmutant lines (*P* ≤ 0.05, error < 0.05), **(d **and** e)**: GO enrichment bubble map of AtHSP20-OE and athspmutant lines differentially expressing *HSP20* gene under high calcium stress

In order to investigate the regulatory mechanism of AtHSP20 in response to high-calcium stress, AtHSP20-OE and athspmutant lines were treated with higcalcium, and the leaves were subjected to transcriptome analyses. The WT was used as the control for identifying the gene sets that were activated in response to high-calcium stress. Hierarchical clustering was performed for generating heatmaps, which revealed that the genes clustered into two groups (Fig. 7a and b). There were significant differences in the expression patterns of certain genes between the AtHSP20-OE and WT lines, indicating that the *HSP20* gene can respond to high-calcium stress in plants. According to the obtained results, compared with WT, 1464 and 129 DEGs were detected in AtHSP20-OE and athspmutant lines, respectively (Fig. 7c). Further analysis showed that there were 100 common DEGs between AtHSP20-OE vs. WT and athspmutant vs. WT (Supplementary Table S1).

To determine the response of AtHSP20-OE and athspmutant lines to high-calcium stress, and identify the biological processes that are affected by the *AtHSP20* gene, the heatmaps of the AtHSP20-OE and athspmutant lines were analyzed by clustering (Fig. 7a and b) and enrichment function of GO using seqenrichment (Fig. 7d and e) were also performed. The results demonstrated that DNA replication and cell proliferation were significantly upregulated in AtHSP20-OE and athspmutant lines under high-calcium stress. These findings revealed that the AtHSP20-OE and athspmutant lines regulated the cells and related genes for resisting the external high-calcium stress. GO enrichment analysis revealed that the number of genes related to abiotic stress response was highest in athspmutant lines, and the expression of related genes encoding galacturan 1-4-alpha-galacturonidase and phosphoglycolate phosphatase was highest in athspmutant lines. In AtHSP20-OE lines, the number of related genes expressed in response to hormone was the highest, and the enrichment of related genes was the highest in the pectin catabolic process. These results indicated that AtHSP20-OE lines, harboring the *HSP20* gene, were capable of regulating different genes for resisting the external high-calcium stress; however, the athspmutant lines that lacked the *HSP20* gene possibly obtained energy via the usual processes that are triggered in response to abiotic stimuli, and via other pathways in the presence of high-calcium stress. The phenotype of AtHSP20-OE lines was significantly superior to that of athspmutant lines under high-calcium stress. Analysis of the physiological and biochemical indices, including the leaf chlorophyll content, revealed that the AtHSP20-OE lines were capable of resisting high-calcium stress, whereas the athspmutant lines exhibited null or negligible resistance to high-calcium stress. In order to further explore the potential control network, we simultaneously conducted DEGs enrichment analysis between these two gene sets and obtained the KEGG pathway map (Fig. 6). The *AT3G30460* gene encodes proteins in the U-box superfamily, and the findings revealed that the *AT3G30460* gene was upregulated by approximately 3.2 fold in AtHSP20-OE lines compared with athspmutant lines. These results indicated that the *HSP20* gene of AtHSP20-OE lines may affect the expression of U-box superfamily proteins via *AT3G30460* to ensure the normal functioning of the ubiquitin 26 S proteosome pathway (UPP), and regulated the selective degradation of plant proteins to maintain the normal growth and development of AtHSP20-OE lines. In addition, U-box superfamily proteins also have certain resistance to salt stress and drought stress. *DREB1A* gene is widely used in plants to resist abiotic stress (such as drought resistance, high salt stress, etc.). The results of transcriptome analysis revealed that the *DREB1A* gene was upregulated in AtHSP20-OE lines by 2.7 fold compared with athspmutant lines. It suggested that in AtHSP20-OE lines, *HSP20* gene affects the expression of *DREB1A* gene to increase at resistance and jointly resist high calcium stress environment. The *BBX18* gene is a member of the BBX family of transcription factors, and the *BBX18* gene was found to be upregulated in AtHSP20-OE lines by 1.5 fold compared to that of athspmutant lines. This finding suggested that the *HSP20* gene may also affect the expression of *BBX18* to enhance its regulatory effect on various physiological processes, including growth, development, and response to abiotic stress, which increased the tolerance of AtHSP20-OE lines to high-calcium stress. These results suggested that the expression of the *HSP20* gene in *A. thaliana* resisted high-calcium stress by modulating the expression of key regulatory genes, including *DREB1A*, *BBX18*, and others, to maintain the normal physiological activities of *A. thaliana*.

## Discussion

Compared with *C. nitidissima*, *C.limonia* as a karst species has the characteristics of high calcium resistance. *C.limonia* can cope with high-calcium stress in karst environments via structural, functional, and physiological modulations resulting from long-term adaptive evolution. However, there are few researches about the mechanism of suitable karst growth of *C. limonia* with specific gene functions, and the regulation mechanism of *HSP20* gene in growth of *C. limonia* in karst environment with high calcium is still unclear.

In this study, the Arabidopsis plants that overexpressed ClHSP20, CnHSP20, and AtHSP20 and the athspmutant lines were exposed to high-calcium stress. The ClHSP20-OE, CnHSP20-OE, AtHSP20-OE, athspmutant, and WT lines showed different high calcium resistance, and the regulation mechanism of *ClHSP20* gene of *C. limonia* in high calcium environment could be systematically obtained. The key differentially expressed genes and the important adaptive regulatory pathways were found to explain the response mechanism and adaptive regulation of *C. limonia* to high calcium stress.

### The change of leaf foliar physiological indices could help *C. limonia* adapt to high calcium mountain environment

The results of this study showed that under high calcium treatment, ClHSP20-OE lines had the highest stomatal pore size, stomatal conductance, and CO_2_ conductance, and the lowest intercellular CO_2_ concentration than WT and athspmutant. Porosity is an important channel for the exchange of water and CO_2_ gas. CO_2_ in the air diffused into the photosynthetic interstitial space of leaves through the stomata, and photosynthesis occurred under the action of light energy. The water simultaneously diffuses through the stomata into the atmosphere via transpiration [41]. Stomatal conductance is an important factor in determining the photosynthetic intensity and rate of transpiration [42]. Previous studies have indicated that stomatal restriction caused by the partial closure of stomata under stress reduces the photosynthetic efficiency of leaves [43]. In this study, the ClHSP20-OE lines were strong ability of exchanging gas and water under high-calcium stress, which ensured the stability of the photosynthetic process. It has been reported that the sensitivity of stomata to alterations in the external environmental is an important indicator of environmental adaptation [44]. With the prolonging of high calcium treatment time, the photosynthetic rate of all Arabidopsis lines presents a decreasing trend, which is similar to the results of Campos [45] and Josephine Wu [46].

The contents of leaf chlorophyll and nitrogen, net photosynthetic rate, and other indices of ClHSP20-OE lines were higher than those of the WT and athspmutant lines under high-calcium stress. The photosynthetic capacity of plants depends on the physiological characteristics of each component of photosynthetic organs. Nitrogen is an important constituent for the synthesis of chlorophyll, and 75% of the nitrogen content of plants is concentrated in chloroplasts, of which the majority is used for the construction of photosynthetic organs. Therefore, the nitrogen content is an important factor in regulating photosynthetic metabolism [47], and the chlorophyll content is fundamentally proportional to the content of leaf nitrogen [48]. Therefore, nitrogen content and chlorophyll content of plant leaves play an important role in plant photosynthesis to ensure normal growth under abiotic stress. The data obtained in this study revealed that the contents of chlorophyll and nitrogen in the leaves tended to decrease in all the plant groups under high-calcium stress. The reduction in the contents of chlorophyll and nitrogen was lowest in ClHSP20-OE lines, compared to those of the CnHSP20-OE, AtHSP20-OE, and athspmutant lines. These results indicated that high-calcium stress can reduce the contents of chlorophyll and nitrogen in the leaves; however, the *ClHSP20* gene suppressed this reduction, which ensured that the contents of foliar chlorophyll and nitrogen remained stable in ClHSP20-OE lines. Xu et al [49] reported that the content of foliar chlorophyll decreases under salt stress, which negatively affects the absorption of light energy and subsequently reduces photosynthesis. Under high calcium treatment, the net photosynthetic rate of all lines showed a downward trend. Compared with WT lines, the net photosynthesis of ClHSP20-OE lines increased by 140.6%, and the decrease degree of ClHSP20-OE lines was smallest in all lines. This data is similar to Xu’s [49] study. Altogether, these results indicated that the *ClHSP20* gene can reduce the decomposition of chlorophyll and increase the nitrogen content under high-calcium stress to ensure the stability of the net photosynthetic rate and photosynthesis, and thus maintain normal plant growth.

The findings additionally revealed that the contents of soil nitrogen, phosphorus, and potassium for ClHSP20-OE lines were higher than those of WT lines, but the contents were lowest for athspmutant lines. Nitrogen, potassium, phosphorus are important elements for plant growth and development, and nitrogen is an important component of plant chloroplasts. Plants take up nitrogen from the soil for photosynthesis and accumulating organic matter, which contribute to the formation of various components, including several proteins, chlorophyll, and ATP. The uptake of nitrogen plays an important role in plant growth and development, foliar gaseous exchange, and carbon metabolism [50]. It is therefore speculated that the phosphorus, potassium, and nitrogen utilization efficiency of ClHSP20-OE lines was more stable, which was beneficial for their survival in a high-calcium environment. These results suggested that the *ClHSP20* gene can respond to high-calcium stress, and may help plants adapt to mountainous environments with high soil calcium levels by enhancing the rates of photosynthesis, transpiration, and nitrogen utilization.

### Reactive oxygen scavenging mechanism may play an important role in response to high calcium stress in *C. limonia*

Reactive oxygen species (ROS) is a form in which oxygen is partially reduced or activated. It is a general term for oxygen containing substances with extremely active properties and strong oxidation ability, including superoxide anion (O_2_^−^), hydrogen peroxide (H_2_O_2_), hydroxyl radical (-OH), etc. ROS are toxic by-products of aerobic metabolism in plants [51], and they can diminish the photosynthetic rate under abiotic stress.

An increase in the contents of ROS, including H_2_O_2_ and O_2_^−^, has adverse effects on plant growth [52]. Superoxide dismutase (SOD) plays an important role in protecting cells from oxygen free radical poisoning [53]. SOD converts O_2_^−^ into H_2_O_2_ via a dismutation reaction, following which catalase and oxidase convert the produced H_2_O_2_ into water to remove the intracellular free oxygen radicals and alleviate plant stress response. [54].

Previous studies have reported that plants with stronger superoxide scavenging abilities can maintain a superior growth status under drought stress [55]. Under high temperature stress, plants can resist the damage caused by peroxidation induced by high temperature stress by increasing the activity of antioxidant enzymes [56]. Therefore, the removal of ROS plays a key role in plant responses to abiotic stresses such as drought, high temperatures, and low phosphorus levels [23]. In this study, the results of DAB and NBT staining revealed that compared with WT lines, the accumulation of H_2_O_2_ and O_2_^−^ in the foliar tissues of ClHSP20-OE lines was lowest under high-calcium stress, followed by AtHSP20-OE and athspmutant lines; however, the accumulation of ROS was highest in CnHSP20-OE lines. It is therefore further speculated that *ClHSP20* aids the adaptation of *C. limonia* to the high-calcium stress in karst areas by enhancing the free radical scavenging ability, and the mechanism of ROS scavenging may play an important role in environmental adaptation.

### Regulation of key genes on biosynthetic metabolic pathways of *C. limonia* in response to high calcium stress

Previous studies have demonstrated that the *HSP20* gene plays an important role in the abiotic stress response of different plants, including sugarcane [23], peanut [57], laver [58], etc. The abiotic stress response induced by *HSP20* is a complex process that is mediated by various factors. Transcriptome analysis revealed that the differential expression of *FBA5*, *AT3G30460*, *DREB1A*, *POL*, *AT5G10770*, and other genes under high-calcium stress affects certain metabolic pathways and the synthesis of metabolites in *Arabidopsis thaliana*.

Fructiose-1,6-bisphosphate aldolases (FBAs) are a common metabolic enzyme that are essential for glycolysis and gluconeogenesis [59]. In higher plants, FBAs also participates in the Calvin cycle and plays an important role in regulating the rate of photosynthesis [60]. It has been reported that the *FBA5* gene plays an important role in plant response to salt stress and related abiotic stresses [60]. Cai et al. reported that the expression of *SlFBA4* and *SlFBA7* is induced by low and high temperature stress. The study additionally revealed that the activity of *FBA* was higher in plants overexpressing *SlFBA4* and *SlFBA7*, and the reduction in their photosynthetic rates was lower, which significantly improved the tolerance of transgenic tomato plants to low temperature and low light stress [61]. In this study, the expression of *FBA5* in ClHSP20-OE lines was upregulated by 9.1 fold compared to that of CnHSP20-OE lines. Phenotypic analysis revealed that the reduction in the net photosynthetic rate of ClHSP20-OE lines was lower than that of the CnHSP20-OE lines. These results indicated that *ClHSP20* gene may increase the expression of *FBA5* gene to maintain a stable photosynthetic rate, and thus improve the high calcium tolerance of *C. limonia.*

The *AT3G30460* gene encodes proteins in the RING/U-box superfamily, and plays a key role in the ubiquitination pathway that mediates the process of protein degradation, and in turn plays an important role in plant disease resistance, stress resistance, growth and development [55]. Additionally, U-box proteins promote the ubiquitination and degradation of *TaIRT1* and *TaIAA17* to regulate plant stress tolerance [62]. It has been reported that the PUB15 Rice U-box protein of rice is also significantly induced under conditions of salt stress. A previous study demonstrated that the oxidative damage caused by high salt conditions reduced significantly in plants overexpressing PUB15, and the plants exhibited strong salt tolerance, indicating that PUB15 can reduce the ROS content and positively regulate the salt stress response [63]. The results of transcriptome analysis in this study revealed that the expression of *AT3G30460* was upregulated by 3.2 fold in AtHSP20-OE lines compared to athspmutant lines. Phenotypic analysis combined with DAB and NBT staining revealed that the intensity of DAB and NBT staining was lightest in ClHSP20-OE lines among all the plant lines, indicating that the content of ROS was lowest in ClHSP20-OE lines. It can be speculated that *AtHSP20* upregulated the expression of *AT3G30460* and aided the adaptation of Arabidopsis to a high-calcium environment by regulating ubiquitin-mediated protein degradation.

Drought response element-binding protein (DR-Responsive Element-Binding Protein) is a class of transcription factors that contain the DNA-binding domain of AP2 [64]. A previous study reported that the H_2_O_2_ content of *DREB1A* transgenic wheat decreased under drought stress compared to that of the WT, indicating that the extent of damage to the cell membrane of transgenic wheat plants exposed to drought stress was lower than that of WT lines, which enabled the transgenic wheat plants to cope better with the drought stress [65]. The results of transcriptome analysis in this study revealed that the expression of *DREB1A* was upregulated by 2.7 fold in AtHSP20-OE lines compared to that of athspmutant lines. Phenotypic analysis combined with DAB staining revealed that the intensity of DAB staining was lowest in ClHSP20-OE lines compared to all the plant lines, indicating that the content of H_2_O_2_ was lowest in ClHSP20-OE lines, which was consistent with the findings of previous studies. These results indicated that *AtHSP20* can promote the expression of *DREB1A* in response to a high-calcium environment.

The *POL* gene is associated with the expression and synthesis of protein phosphatases 2 C (PP2C). As an important regulator of the ABA signaling pathway, PP2C functions as a kind of multifunctional constitutive protein phosphatase and plays a key roles in plant growth and development, hormone signaling, and environmental stress response [66]. Numerous studies have demonstrated that the *POL* gene of plants can negatively regulate ABA signaling pathways and various stress response pathways. demonstrated that the *POL* gene is negatively regulated during the drought stress response of *Arabidopsis thaliana.* [67]. It has been reported that ZmPP2C functions as a negative regulator salt and drought stress responses in plants [68]. The PP2C31 protein of *Arabidopsis thaliana* is a non-abscisic acid-dependent negative regulatory component that is induced in response to high salt stress [69]. In this study, the results of transcriptome data analysis revealed that the expression of the *POL* gene was down-regulated in AtHSP20-OE lines by 9.6-fold under high-calcium stress, compared to that of athspmutant lines. It is speculated that the *AtHSP20* gene can negatively regulate the response to high-calcium stress by regulating the *POL* gene.

Aspartic proteases (APs) encoded by the *AT5G10770* gene play an important role in plant responses to abiotic stresses, including drought, UV, salicylic acid, and cold injury [70]. It has been reported that exposure to drought stress can increase the expression levels of AP in guard cell 1 (ASPG1) in Arabidopsis, and the overexpression of ASPG1 enhances the ABA sensitivity of stomatal guard cells. ASPG1 enhances plant adaptation to drought stress by partaking in ABA signaling in guard cells [71]. Stomatal restriction caused by the partial closure of stomata can reduce the photosynthetic efficiency of leaves [43]. Therefore, the rate of stomatal conductance plays an important role in the normal growth of plants. The results of transcriptome analysis in this study revealed that the expression of *AT5G10770* in ClHSP20-OE lines was up-regulated by 2.6 fold compared to that of CnHSP20-OE lines. The results of transcriptome and phenotypic analyses revealed that the stomatal conductance and photosynthetic rate of ClHSP20-OE lines were higher than those of CnHSP20-OE lines. It is speculated that the *ClHSP20* gene upregulated the expression of the *AT5G10770* gene, which affected ABA signal transduction in stomatal guard cells and promoted the adaptation of Arabidopsis to high-calcium stress.

Calcium ions act as secondary messengers in plant cells during signal [72]. Calcium regulates numerous physiological and biochemical reactions in plants, and serves as a vital metabolic regulator while playing an essential role in plant growth and development [73]. However, a high concentration of calcium in soils can cause osmotic stress, interfere with ion balance, inhibit plant growth and development, and even result in plant death [74]. However, certain plants are calcium-tolerant and are thus able to tolerate the relatively high concentrations of soil calcium. Resistance to the high levels of calcium in karst regions has enabled plants to grow despite high-calcium levels and develop novel regulatory mechanisms. Analysis of the functions of the *HSP20* gene of *C. limonia* in mediating calcium tolerance revealed that *HSP20* genes affect plant growth and regulate gene expression. Results revealed that the expression levels of certain key influencing factors, including *FBA5* and *AT5G10770*, differed significantly between CnHSP20-OE and ClHSP20-OE lines, and the expression levels of *DREB1A* and *AT3G30460* differed significantly between AtHSP20-OE and athspmutant lines, indicating that the mechanism of calcium tolerance of plants is complex and regulated by multiple genes. Additionally, these key DEGs may play important roles in the response to high-calcium stress, and further studies on these DEGs are necessary for generating resistance to high-calcium stress. The findings obtained herein provides insights for future studies on the protection of rare and endangered plants, and also identified a potential candidate reference gene for studying the calcium resistance of other plants.

## Conclusion

To elucidate the possible mechanisms underlying the adaptation of yellow Camellia to high levels of environmental calcium, the *HSP20* gene of yellow Camellia was initially studied here. The *ClHSP20*, *CnHSP20*, and *AtHSP20* genes were overexpressed in *Arabidopsis thaliana*, and athspmutant lines with *AtHSP20* gene knockout were used for analysis. All the lines were treated with high calcium to detect their high calcium resistance. The findings revealed that the ClHSP20-OE lines exhibited the highest resistance to high-calcium stress, whereas athspmutant lines exhibited the weakest resistance. The resistance of CnHSP20-OE and AtHSP20-OE lines ranged between that of CIHSP20-OE and athspmutant lines.

In order to further elucidate the adaptive regulation of yellow Camellia growing in the high-calcium karst environments at the transcriptional level, the transcriptome data of the plants were analyzed. The results demonstrated that exposure to high-calcium levels activated more genes in ClHSP20-OE lines, and the expression levels of these genes increased in response to high-calcium stress. The expression levels of *FBA5* and *AT5G10770* in ClHSP20-OE lines increased significantly compared t with CnHSP20-OE lines. The expression levels of *POL*, *AT3G30460*, *DREB1A*, *BBX18*, and other genes differed significantly between AtHSP20-OE and athspmutant lines. The findings revealed that *FBA5*, *AT5G10770*, *POL*, *AT3G30460* and *DREB1A* genes played an important role in the response to high-calcium stress. These results suggest that *HSP20* gene, together with other factors, plays an important role in plant adaptation to high-calcium environment.

### Electronic supplementary material

Below is the link to the electronic supplementary material.

Supplementary Material 1 Supplementary Material 1

Supplementary Material 2

Supplementary Material 3 Supplementary Material 3

Supplementary Material 4 Supplementary Material 4

Supplementary Material 5

Supplementary Material 6

Supplementary Material 7

Supplementary Material 8

## Data Availability

The raw sequencing data have been deposited to the NCBI (https://submit.ncbi.nlm.nih.gov) with Sequence Read Archive (SRA) accession No. PRJNA987132.
