# Supplementary figures and images for: Heterologous overexpression of heat shock protein 20 genes of different species of yellow Camellia in Arabidopsis thaliana reveals their roles in high calcium resistance
Source: BMC Plant Biol. 2024 Jan 2;24:5. doi: 10.1186/s12870-023-04686-x (PMC10759694; doi:10.1186/s12870-023-04686-x)

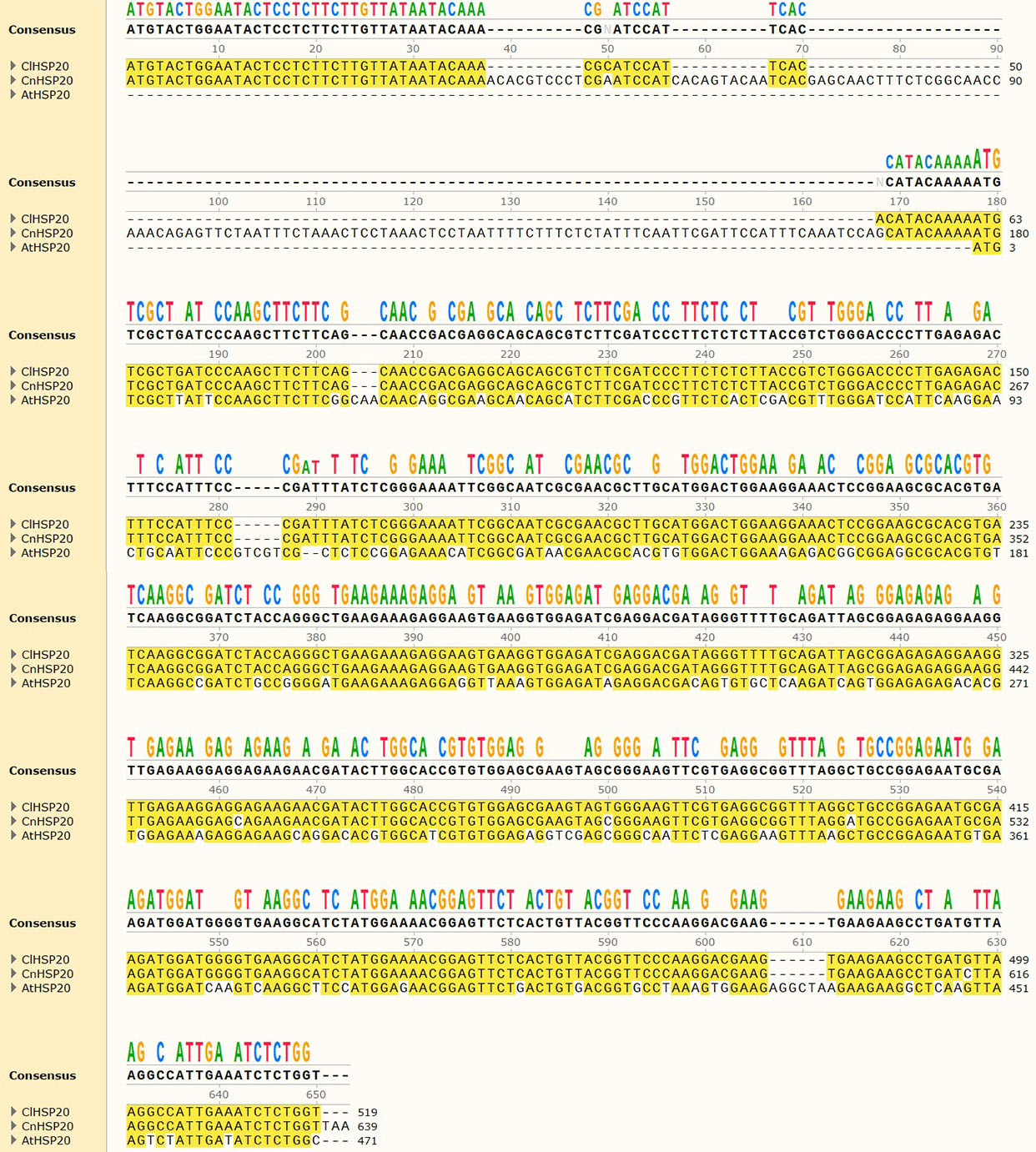

Supplement: Supplementary file 1 — Supplementary Material 1 [file 12870_2023_4686_MOESM1_ESM.png]

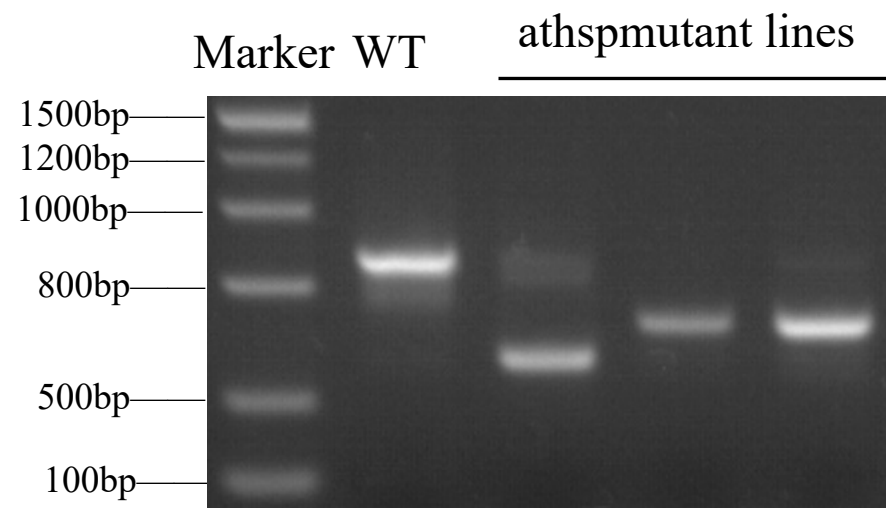

Supplement: Supplementary file 2 — Supplementary Material 2 [file 12870_2023_4686_MOESM2_ESM.pdf]

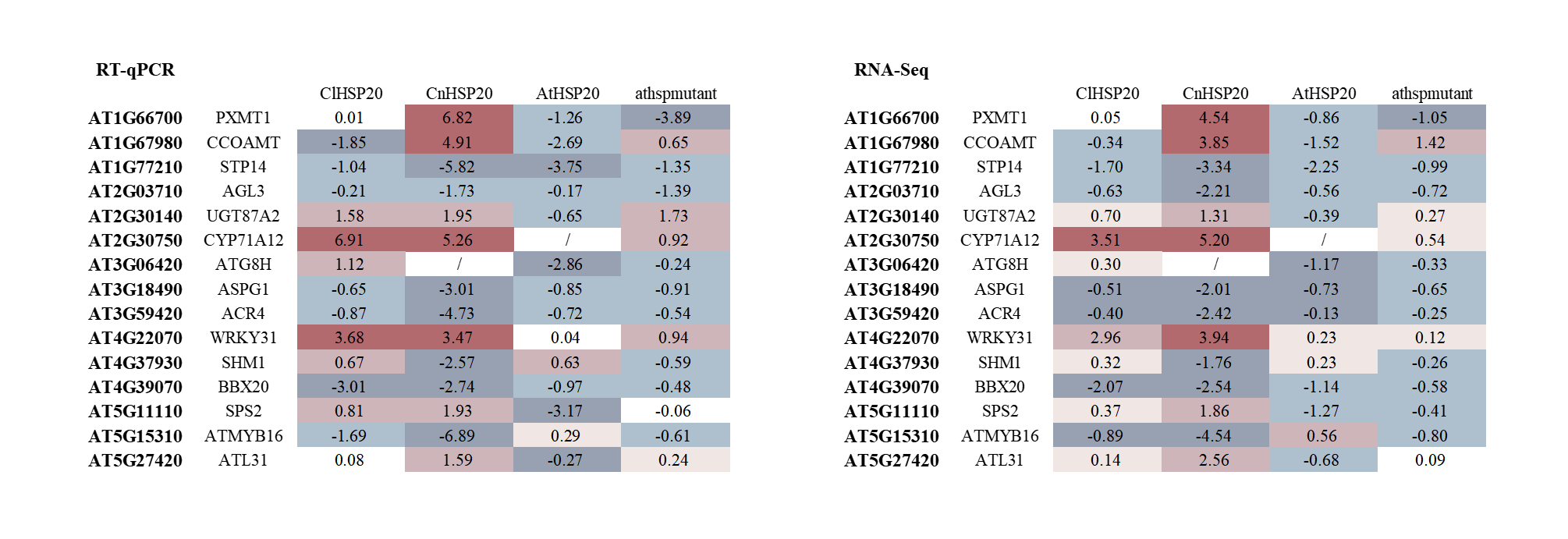

Supplement: Supplementary file 3 — Supplementary Material 3 [file 12870_2023_4686_MOESM3_ESM.png]

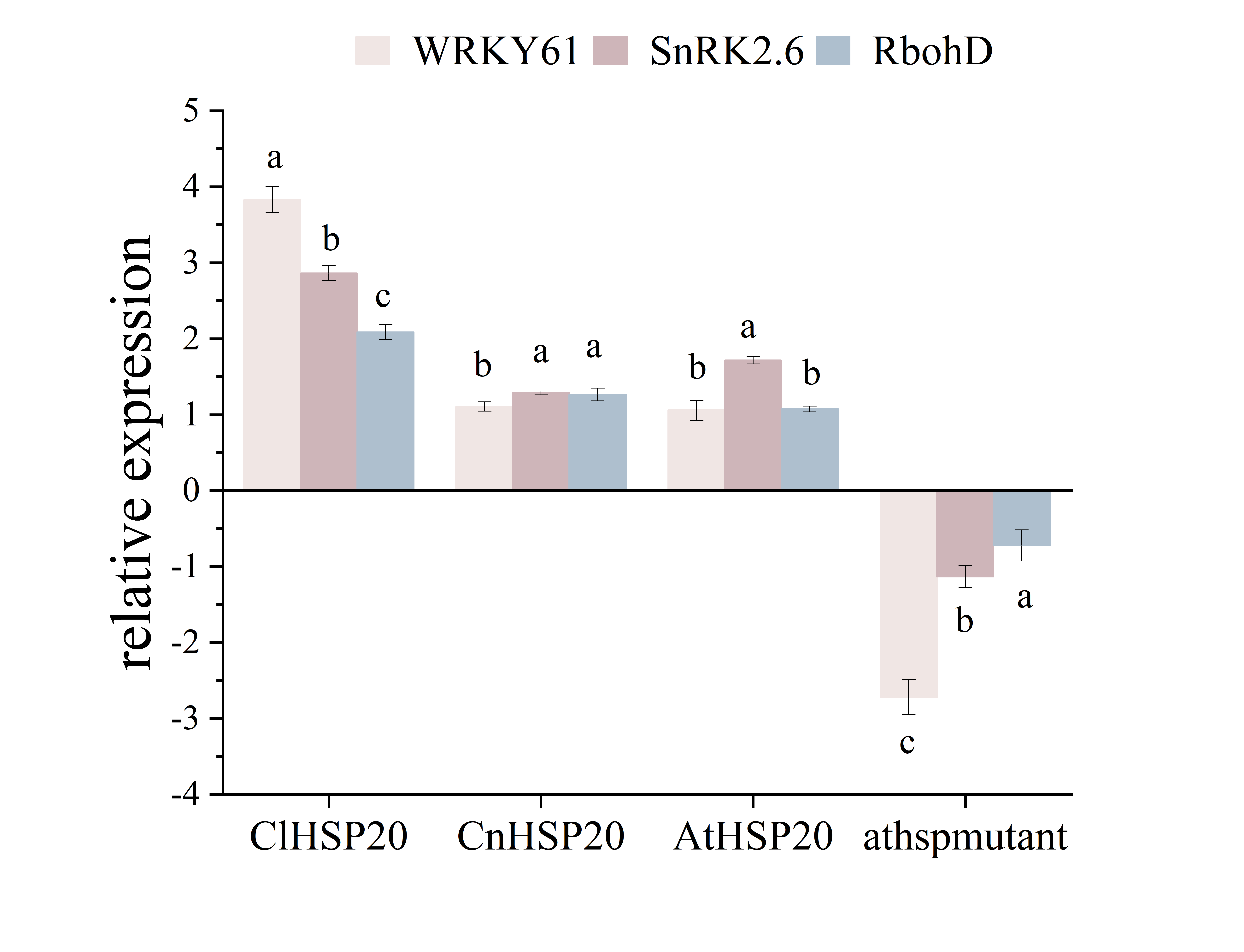

Supplement: Supplementary file 4 — Supplementary Material 4 [file 12870_2023_4686_MOESM4_ESM.png]
