# Supplementary material for: Heterologous overexpression of heat shock protein 20 genes of different species of yellow Camellia in Arabidopsis thaliana reveals their roles in high calcium resistance
Source: BMC Plant Biol. 2024 Jan 2;24:5. doi: 10.1186/s12870-023-04686-x (PMC10759694; doi:10.1186/s12870-023-04686-x)
Supplement: Supplementary file 8 — Supplementary Material 8 [file 12870_2023_4686_MOESM8_ESM.pdf]

Supplementary Figure Legends

Supplementary Figure S1

Sequence alignment of the *HSP20* genes in CnHSP20-OE, ClHSP20-OE, AtHSP20-OE, athspmutant and WT lines.

Supplementary Figure S2

The full-length gel of PCR confirmation results of athspmutants. The lanes from left to right:1: Marker, 2: WT, 3-5: athspmutant lines.

Supplementary Figure S3

Comparison of the results between RT-qPCR and RNA-seq.

Supplementary Figure S4

Relative expression of abiotic stress-related marker genes.

Supplementary Table S1

AtHSP20-OE vs WT and athspmutant vs WT common DEGs.

Supplementary Dataset File S1

The key differentially expressed genes between ClHSP20-OE lines and CnHSP20-OE lines.

Supplementary Dataset File S2

The key differentially expressed genes between AtHSP20-OE lines and athspmutant lines.
